# Behavioral Studies Using Large-Scale Brain Networks – Methods and Validations

**DOI:** 10.3389/fnhum.2022.875201

**Published:** 2022-06-16

**Authors:** Mengting Liu, Rachel C. Amey, Robert A. Backer, Julia P. Simon, Chad E. Forbes

**Affiliations:** ^1^School of Biomedical Engineering, Sun Yat-sen University, Shenzhen, China; ^2^Department of Psychological and Brain Sciences, University of Delaware, Newark, DE, United States; ^3^Keck School of Medicine, University of Southern California, Los Angeles, CA, United States; ^4^Department of Psychology, Florida Atlantic University, Boca Raton, FL, United States

**Keywords:** neuroscience, graph theory, data driven, machine learning, neural network

## Abstract

Mapping human behaviors to brain activity has become a key focus in modern cognitive neuroscience. As methods such as functional MRI (fMRI) advance cognitive scientists show an increasing interest in investigating neural activity in terms of functional connectivity and brain networks, rather than activation in a single brain region. Due to the noisy nature of neural activity, determining how behaviors are associated with specific neural signals is not well-established. Previous research has suggested graph theory techniques as a solution. Graph theory provides an opportunity to interpret human behaviors in terms of the topological organization of brain network architecture. Graph theory-based approaches, however, only scratch the surface of what neural connections relate to human behavior. Recently, the development of data-driven methods, e.g., machine learning and deep learning approaches, provide a new perspective to study the relationship between brain networks and human behaviors across the whole brain, expanding upon past literatures. In this review, we sought to revisit these data-driven approaches to facilitate our understanding of neural mechanisms and build models of human behaviors. We start with the popular graph theory approach and then discuss other data-driven approaches such as connectome-based predictive modeling, multivariate pattern analysis, network dynamic modeling, and deep learning techniques that quantify meaningful networks and connectivity related to cognition and behaviors. Importantly, for each topic, we discuss the pros and cons of the methods in addition to providing examples using our own data for each technique to describe how these methods can be applied to real-world neuroimaging data.

## Introduction

A key challenge in cognitive neuroscience is determining how human behaviors or mental representations map onto patterns of neural activity. Research traditionally hypothesizes that specific human behavior or cognitions are closely associated with the activity of a single brain region. Modern neuroscience methods, specifically the development of fMRI, have expanded the human neuroimaging scope from identifying regional activation in brain images to communication between pairs of brain regions (Mill et al., 2017). However, evidence now overwhelmingly indicates that whole-brain functional and network activations can be indexed to provide insight into the mechanisms behind human behaviors.

Whole-brain networks play a fundamental role in neuroscience, and numerous scientists have been fascinated by their ability to reveal the brain’s intricate functional properties. Whole-brain networks identify neural connectivity in a relatively unbiased manner. That is, one must differentiate the meaningful signatures of neural activity that underlie behaviors from noisy or redundant neural activity. Setting up reliable functional connections, or brain network-based neuromarkers, is pivotal in investigating human behaviors. However, selecting the best neuromarkers in relation to behaviors from the intensive whole-brain network is difficult.

Measuring whole-brain neural network activity is complex. It has been suggested that the brain acts as a parallel processor, meaning that multiple regions influence one another across whole-brain neural networks simultaneously. Associations between these whole-brain neural connections and human behavior often require multi-level approaches that have the potential to include every single connection, regional organization, and the whole-brain topological architecture. The noisy nature of brain activity, let alone whole brain activity, requires a more sensitive statistical approach to detect the robust associations with behaviors. Furthermore, recent theories have emphasized that neural computation may be more dynamic than previously thought (Langdon and Chaudhuri, 2021). In other words, networks previously associated with behavior may need to be reconsidered in a more dynamic fashion. With the help of recent advances in statistical methods it is now much easier to find clear brain-behavior associations from unbiased whole-brain networks in static and dynamic time series. However, given the complexity of the results, meaningful interpretations of identified brain regions can be a challenge. Selecting an optimal technique is integral for the interpretation of results.

This review focuses on two topics: First, how analyzing whole-brain neural networks can facilitate our understanding of neural mechanisms and build models of behavior. Second, this review focuses on implementing a multi-level approach, both spatially and temporally, to obtain unbiased whole-brain neural network results. We review popular approaches to analyze whole-brain activity such as graph theory, connectome-based predictive modeling, multivariate pattern analysis, network dynamic modeling, and deep learning techniques that quantify meaningful whole-brain networks regarding cognition and behavior. Importantly, each technique will be reviewed with pros and cons of its application to inform readers of the best approach for their data (a table is also provided for convenience, Table 1). Furthermore, we also provide examples for each technique, describing how the method can be applied to real-world neuroimaging data.

**TABLE 1:** The strength and weakness of each method in behavioral prediction scenarios.

Method	Application scenarios	Strengths	Weaknesses
Graph theory	To quantify human behaviors using the brain network’s topological architecture or organization.	Allows researchers to quantify multiple regions to describe neural network architecture in myriad ways (e.g., efficiency, connectivity strength)	Often, the interpretation of global or regional graph theoretical measures in relation to human behaviors is ambiguous. Causality can not be inferred.
CPM	To search for all neural connections in relation to human behaviors using a data-driven approach.	Allows researchers to examine neural activity related to behavior without any prior bias. Novel connections are often found in relation to behavior.	The functional connectivity revealed may not conventionally relate to the examined behavior. Researchers must take care in the interpretation of the results and often replication or validation is needed.
MVPA classification	To increase the sensitivity of recognizing behavior related cognitive states in human brain network.	Allows researchers to focus on the statistical nature of mental representations via classification by activation similarities.	Creating an optimal predictive model using MVPA in order to finding cognitively meaningful features needs to be further validated regarding neural networks.
MVPA RSA	To identify how similarly/differently human brain network performs in different cognitive states.	Allows researchers to focus on the statistical nature of mental representations via classification by representational distance, e.g., dissimilarities.	The association with behavioral scores is not straightforward. These associations must be interpreted carefully after considering possible cognitive theories.
Brain network dynamics	To evaluate the moment-to-moment variability of brain network representations in relation to behaviors.	Allows researchers to utilize time series to provide insight into how neural networks fluctuate in a temporal manner in relation to cognitive processes and behavior.	Too many connections in neural networks may lead to ineffective time series segmentation. Feature reduction is needed before dynamic modeling.
GCN based deep learning	To significantly increase the prediction accuracy for behavioral performance scores in “big data” scenarios using brain network.	Allows researchers to estimate incredibly accurate behavior predictions from neural activation if the sample size is sufficient.	Because of the large number of parameters within deep learning models, these models can often overfit in small samples. Indeed, GCN models not only need connectivity values but also the features on nodes within neural networks. The added features differentiate this analysis from other conventional network analyses which are solely based on neural connections. Thus, the cognitive interpretation is difficult because the model is often too complicated to localize the features that contribute to the prediction results.
Meta-analysis	To pre-select a related functional connectivity in hypothesis-driven behavioral studies.	Allows researchers the opportunity to leverage past literature to bolster current studies. Also provides a tool to help replicate past findings.	Researchers lose the opportunity to identify novel findings.

## Method

In this paper, we will use our own data to demonstrate the usage of the above-mentioned data-driven approaches for whole-brain analyses, illustrating how whole-brain data-driven approaches can provide additional insight into cognition studies.

### General Procedure

Sixty-five participants (33 males) came into the lab to complete two cognitive tasks. The first was a problem-solving task consisting of math problems and performance feedback. The second was a memory test that indexed what participants saw during the problem-solving task. Live electroencephalogram (EEG) recordings were completed during the entire experimental session. Participants were seated in a sound proofed chamber, set up with an EEG, and were instructed to begin the task which was displayed on a computer screen in front of them. Participants were able to move through the task by pressing buttons on a button box placed in their laps. The present set up minimizes movement from the participant which can contribute to noisy neural data. The problem-solving task consisted of a 34-min math task consisting of standard multiplication and division problems (e.g., 10 × 20 =) that initial pilot tests confirmed varied in degree of difficulty to ensure all participants would solve problems correctly and incorrectly. During each trial, participants were given three answer choices below each problem (A, B, or C) presented on the screen, with the answer to each problem randomly presented in one of the three answer positions on each trial. Participants made all answer selections via the button box placed in their laps and did not have scratch paper or a calculator. After each response, participants received feedback for 2 s that indicated whether their selected answer to the math problem was wrong or correct. To assess memory for feedback (indexed in the second task), the words “Wrong” or “Correct” were presented in a novel font on every trial (see Forbes et al., 2015; Forbes et al., 2018 for examples). Participants were given 16 s to solve each problem. If participants were unable to answer a problem within that time frame, they would receive negative feedback (i.e., “Wrong” would appear on the screen). Participants completed an average of 83.9 problems. The present data is ideal for this review as it ensured participants went through myriad cognitive processes while live EEG was recorded. Furthermore, the repetitive nature of the math and memory tests (further described below) ensured we would have enough trials per stimuli to test these advanced methods.

### Memory Test

Like Forbes et al. (2018), participants were presented with a surprise memory test containing 400 trials after the problem-solving task while EEG data was recorded simultaneously. Among the 400 trials, participants were presented with each font/feedback pairing they had previously seen during the problem-solving task, i.e., each performance feedback stimulus that was shown for 2 s, with the remaining trials acting as “lures.” During each trial, participants were randomly presented with the words “wrong” or “correct” written in one of the 200 different fonts in the middle of a computer screen. A scale was presented below each font/feedback combination and participants were asked to indicate whether they had seen the combination during the problem-solving task using a six-point scale (1 = I know I didn’t see it, 4 = I think I saw it, 6 = I know I saw it). If participants were presented with a previously seen font, responses of 4–6 were classified as hits, and responses of 1–3 were classified as misses. If participants were presented with a novel font, responses of 4–6 were classified as false alarms, and responses of 1–3 were classified as correct rejections. Using these classifications, we calculated d-prime scores to measure participants’ ability to accurately discriminate seen from unseen fonts. Prior research suggests that d-prime score is a more sensitive assessment of memory effects that accounts for guessing (Wickens, 2001). To calculate d-prime scores, *z* scores for false alarm rates were subtracted from *z* scores for hit rates. Because *z* scores for 0 or 1 cannot be calculated, participants without hits were given scores of 0.1 and participants with perfect scores were given a score of 0.9. Therefore, larger d-prime scores indicate that participants were better at discriminating between previously seen fonts and lures, i.e., had more accurate memory recall. Within the results presented in this review, d-prime will serve as a proxy for memory accuracy.

### Electroencephalogram Recording

Continuous EEG activity was recorded using an ActiveTwo head cap and the ActiveTwo Biosemi system (BioSemi, Amsterdam, Netherlands). Recordings were collected from 128 Ag-AgCl scalp electrodes and from bilateral mastoids. Two electrodes were placed next to each other 1 cm below the right eye to record startle eye-blink responses. A ground electrode was established by BioSemi’s common Mode Sense active electrode and Driven Right Leg passive electrode. EEG activity was digitized with ActiView software (BioSemi) and sampled at 2,048 Hz. Data was downsampled post-acquisition and analyzed at 512 Hz.

### Electroencephalogram Preprocessing

For performance feedback analyses, the EEG signal was epoched and stimulus locked from 500 ms pre-feedback presentation to 2,000 ms post-feedback presentation. For memory test analyses, EEG signal was epoched and stimulus locked from 500 ms pre-performance feedback presentation (previously seen font/feedback combinations or lures) to 1,000 ms post-feedback presentation. EEG artifacts were removed via FASTER (Fully Automated Statistical Thresholding for EEG artifact Rejection) (Nolan et al., 2010), an automated approach to cleaning EEG data that is based on multiple iterations of independent component and statistical thresholding analyses. Specifically, raw EEG data was initially filtered through a band-pass FIR filter between 0.3 and 55 Hz. Then, EEG channels with significant unusual variance (absolute z score larger than 3 standard deviations from the average), mean correlations with other channels, and Hurst exponents were removed and interpolated from neighboring electrodes using a spherical spline interpolation function. EEG signals were then epoched and baseline corrected; epochs with significant unusual amplitude range, variance, and channel deviation were removed. The remaining epochs were then transformed through ICA. Independent components with significant unusual correlations with EOG channels, spatial kurtosis, slope in the filter band, Hurst exponent, and median gradient were subtracted and the EEG signal was reconstructed using the remaining independent components. In the last step, EEG channels within single epochs with significant unusual variance, median gradient, amplitude range, and channel deviation were removed and interpolated from neighboring electrodes within the same epochs.

### Source Reconstruction

All *a priori* sources used in network connectivity analyses were identified and calculated via forward and inverse models utilized by MNE-python (Gramfort et al., 2013, 2014). The forward model solutions for all source locations located on the cortical sheet were computed using a 3-layers boundary element model (BEM) (Hamalainen and Sarvas, 1989) constrained by the default average template of anatomical MNI MRI. Cortical surfaces extracted with FreeSurfer were sub-sampled to approximately 10,240 equally spaced vertices on each hemisphere. The noise covariance matrix for each individual was estimated from the pre-stimulus EEG recordings after preprocessing. The forward solution, noise covariance and source covariance matrices were used to calculate the dynamic statistical parametric mapping (dSPM) estimated inverse operator (Dale et al., 1999, 2000). The inverse computation was done using a loose orientation constraint (loose = 0.2, depth = 0.8) (Lin et al., 2006). Using depth weighting and noise normalization approaches, dSPM inverse operators have been reported to help characterize distortions in cortical and subcortical regions, and improve the bias accuracy of neural generators in deeper structures, e.g., the insula (Attal and Schwartz, 2013). The cortical surface was divided into 68 anatomical regions (i.e., sources) of interest (ROIs; 34 in each hemisphere) based on the Desikan–Killiany atlas (Desikan et al., 2006) and signal within a seed voxel of each region was used to calculate the power within sources and phase locking (connectivity) between sources.

### Functional Connectivity Estimation and Network Construction

Frequency coupling was calculated within identical frequency bands and temporal periods between all pairs of nodes. Phase locking values (PLV) (Lachaux et al., 1999), which measure variability of phase between two signals across trials, were utilized to define connectivity strength. In other words, for every participant, condition, and frequency band, we obtained a symmetric 68 × 68 adjacency matrix, representing all pairs of nodes – or *edges* – in each participant’s whole-brain network during a given period. For the memory task period, PLVs were averaged from the first 500 ms after the memory task appeared. For the resting state period, PLVs were averaged from the first 500 ms after the onset of the initial fixation cross.

## Graph Theory

**FIGURE 1:**
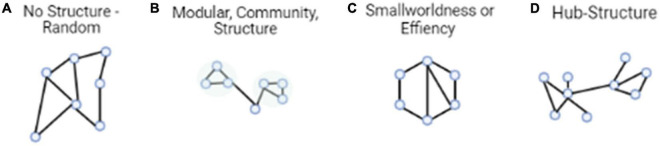
Typical architectural features of functional brain networks. **(A)** The simplest model is entirely random structure. **(B)** Networks with modular structure, divided into communities with dense connectivity. **(C)** Small-world networks, which balance efficient communication and high clustering. **(D)** Networks with hub structure, characterized by a heavy-tailed degree distribution.

Graph theory is the one of the earliest attempts to manipulate large-scale brain networks in cognitive studies. Graph theory allows researchers to integrate multiple regions in an analysis to describe neural network architecture using a global view. As mentioned previously, in the realm of social and behavioral neuroscience, neural activity has often been conceptualized by investigating region-based activity. Graph theory, however, allows one to capture a more wholistic description of the brain by observing the connectivity and neural architecture between regions either in a specific *a priori* network, or across the whole brain, instead of focusing on one area specifically. Within graph theory, modularity, efficiency, and network hubs are standard measures to observe the underlying neural architecture behind various cognitive states and behaviors. We break these measures down below (Figure 1).

### Community Structure

Functional segregation within whole-brain networks and subnetworks plays an essential role in the representation of cognitive states and can be defined by modularity. Modularity quantifies the amount of densely connected nodes, or modules, within a network (Girvan and Newman, 2002; Bullmore and Sporns, 2009). Modules in functional brain networks are thought to represent groups of brain regions that are collectively involved in one or more cognitive domains. Importantly, regions that are anatomically or functionally close to one another are likely to be members of the same cluster or module and share information with one another.

Quantifying neural activity within modularity allows researchers to operationalize neural configurations across the brain. Modularity is often calculated based on hierarchical clustering. In this case, smaller groups of nodes are organized into larger clusters maintaining a scale-free topology (Girvan and Newman, 2002). If a network has high modularity, it can be said to be more functionally segregated, i.e., a subnetwork of nodes within a given network has higher connectivity within itself than it does with the rest of the network (Kim et al., 2020). However, how this network is segregated in relation to the rest of the brain is not quantified by this measure of modularity. Usually, a brain network module that is psychologically meaningful (e.g., working memory network) can be widely distributed among several anatomical modules across the brain.

### Efficiency

Complex whole-brain networks promoted by higher modularity are often more stable and synchronous (Papo et al., 2014). Another important characteristic of cognitive states that can be defined by graph theory is efficiency, or the high output of information transfer with a low connection cost between nodes (Stanley et al., 2015; Cohen et al., 2016). High global efficiency varies between cognitive states and has ramifications on numerous cognitive processes. For example, increased efficiency has positive outcomes on processes such as working memory and spatial orientation ability (Stanley et al., 2015). In these cases, greater neural network efficiency led to both increases in working memory and spatial orientation ability. Greater efficiency has also correlated to better executive task performance and intelligence (Bassett et al., 2009; Li et al., 2009; Van Den Heuvel et al., 2009). These studies all demonstrate the importance of efficiency in representing high functioning cognitive states.

There are a few ways to operationalize efficiency (Rubinov and Sporns, 2010). The most common way to operationalize efficiency is small-worldness. Small-worldness defines a network that is highly clustered, but has short characteristic path lengths (Bullmore and Bassett, 2011). This small-world like structure gives networks a unique property, as they have regional specialization and efficient information transfer across broader regions. If a whole-brain network has a high degree of small-worldness, one would then be able to infer that although it has regional segregation, whole-brain information transfer is still efficient. We can gauge the network efficiency by analyzing *global network efficiency* (GNE) as well. Global network efficiency is a graph theory-based measure that offers perspective about complex mental tasks that we expect to elicit widespread reorganization in the brain (Forbes et al., 2018), i.e., whole-brain reorganization. During cognitively demanding processes requiring more reciprocal communication between remote, specialized areas, an efficient network organization may dynamically facilitate better coordination.

### Network Hubs

All cognitive states require the integration of distributed neural activity across the whole brain. However, it is often the case that specific nodes within these neural networks drive this activity. Utilizing graph theory, analyses can identify these key network hubs that are essential for neural communication and integration. Understanding these hubs provides essential information about the underpinnings of complex cognitive states; functional segregation and specialization are essential for cognitive function. Two types of hubs are essential in describing these cognitive states. For example, if one is interested in one subnetwork in the brain, they could utilize provincial hubs. Provincial hubs are hubs that are mainly connected to nodes within their own network modules. On the other hand, if one was more interested in whole-brain states, one could examine connector hubs. Connector hubs are regions that are highly connected to nodes in other network modules, speaking more to whole-brain connectivity (Guimera and Amaral, 2005). Numerous studies have conducted analyses that note the existence of specific sets of hub regions in various cognitive states, and brain developmental stages (Xu et al., 2021). Specifically, for global communication processes, the precuneus, insular superior parietal, and superior frontal regions have been cited as essential network hubs integral to multiple cognitive processes (Iturria-Medina et al., 2008). Observing network hubs from resting state brain activity has also provided evidence to suggest that communication within the human brain is not random, but rather it is organized to maximize efficient global processing. It is also found that the most pronounced functional connections are found between network hubs that share a common function (Honey et al., 2009).

### Pros and Cons to Graph Theory

Graph theory is an invaluable tool when it comes to quantifying the brain’s network architecture in relation to cognition and behavior by measuring modularity, efficiency, and network hubs. For example, graph theory can allow us to obtain a global, or whole-brain, view of the brain’s configuration during a given cognitive task, providing deeper understanding regarding specific network and regional measures. Given the highly adaptive nature of brain network organization to task demands, this not only indicates the extent to which processes draw on multiple functional organizations, but also provides information about the actual role of the individual communities or sub-networks themselves. Yet these measures still have their limitations. One critical limitation is that the neuro-cortical interpretation of global graph theoretical measures is ambiguous, especially when researchers associate them with human behavioral or cognitive task scores. For example, results often can suggest that one type of stimulus may be more associated with a global network measure over other types. The reasons behind these associations are often unclear; one cannot determine which brain regions are more efficiently connected to others and which regions are not. Even for subjects with equivalent brain network efficiencies, biases toward specific stimuli may be caused by different network organizations. Thus, comparing global measures is not ideal for determining the cognitive mechanisms behind stimuli bias. However, it may be more useful to further contextualize broader graph theory findings by supplementing them with other network analysis methods that target activity in smaller networks, regions, or relationships with behavior.

### Real Data Example

Memory retrieval draws upon multiple functional processes (vision, memory, reasoning, etc.). Because memory retrieval relies on multiple functional processes, it can be expected that more brain regions would communicate with one another during memory retrieval than at rest. Thus, we hypothesized that memory retrieval tasks (remembering unique performance feedback previously displayed during the problem-solving task) would elicit greater *global network efficiency* in connection with retrieval accuracy (i.e., a more efficient brain network would support better memory performance). Global network efficiency was operationalized using small-worldness which quantifies whole-brain neural efficiency. Linear regressions were conducted on memory d-prime scores, operationalizing memory accuracy as stated in the methods, and small-worldness. Significant effects were found between small-worldness and d-prime scores for feedback fonts during the retrieval task for all frequency bands (Theta: β = 1.33, *F*[1,70] = 4.50, *R*^2^ = 0.06, *p* = 0.027; Alpha: β = 1.95, *F*[1,70] = 7.73, *R*^2^ = 0.10, *p* = 0.007; Beta: β = 1.92, *F*[1,70] = 5.92, *R*^2^ = 0.08, *p* = 0.017; Gamma: β = 1.62, *F*[1,70] = 4.98, *R*^2^ = 0.07, *p* = 0.027). All frequency bands demonstrated a positive relationship suggesting the more global efficiency was present, the better memory recall was. This finding illustrates our hypothesis that global network efficiency may positively support cognitive task performance.

## Connectome-Based Predictive Modeling

Setting up a reliable neural-behavior relationship is pivotal in modern cognition studies. Graph theory only provides a “qualitative” evaluation between brain network organizations and human behaviors. In other words, graph theory investigates brain networks from the perspective of topological organizations. However, it is often more important to understand exactly which communications, between pairs of brain regions, contribute to cognitive functions. To date, the establishment of reliable neural-behavior relationships is challenging and a prominent question (Poldrack, 2010; Barch et al., 2013) in neuroimaging studies. Connectome predictive modeling (CPM) can provide insight.

Connectome predictive modeling (CPM; Shen et al., 2017) can reveal the nuances of activity within subnetworks and across the whole-brain using fully data-driven analyses that allow researchers to examine neural activity related to behavior without any prior bias, i.e., predefined brain regions based past literature (see the description of whole brain meta-analyses in the Supplementary Material for further detail). This approach also provides the opportunity to find relationships between novel connectivity and behavioral scores by exploring every single connection within the whole-brain network.

Connectome predictive modeling is particularly useful in predicting human behavior scores. The first step in CPM is to examine each functional connection between all brain regions from the whole-brain network to observe whether they correlate with behavior scores. Often scores that reach a *p* = 0.001 threshold are suggested to be meaningful for this initial step (Rosenberg et al., 2016). Next, a linear model is built to maximize the fit between the summation of selected functional connectivity values and behavior scores. In the last step, CPM uses the linear model to predict behavior scores in new individuals. Currently, CPM doesn’t depend on sophisticated mathematical measures. Instead, CPM discovers meaningful patterns by using simple linear regression models. Because of this approach, it is especially helpful for psychologists, neuroscientists, and clinicians (Cheng et al., 2021) who may have limited background knowledge of more complicated quantitative approaches such as multi-variant pattern analysis (MVPA) and machine learning techniques.

**FIGURE 2:**
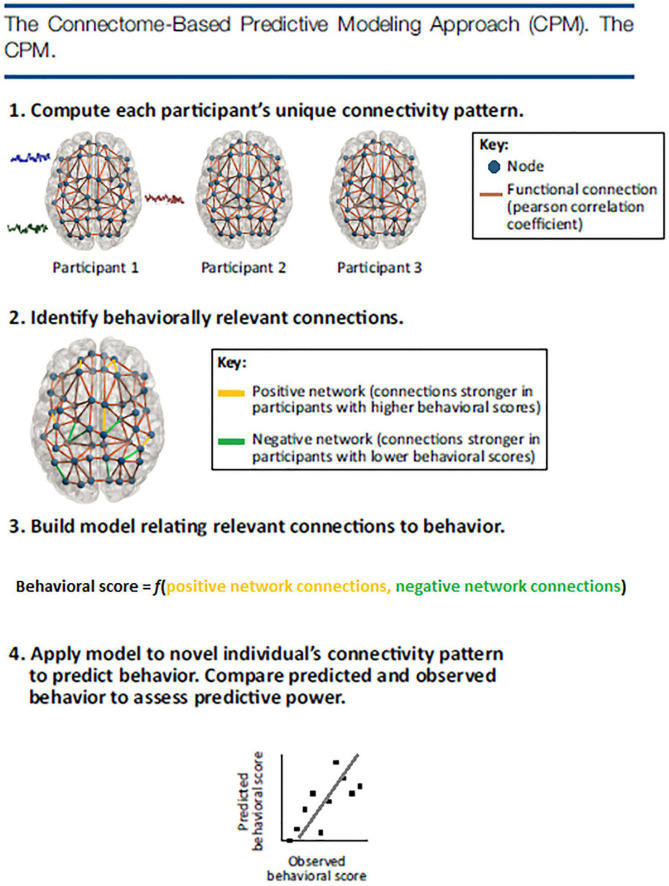
The CPM approach identifies functional connectivity networks that are related to behavior and measures strength in these networks in previously unseen individuals to make predictions about their behavior. First, every participant’s whole-brain connectivity pattern is calculated by correlating the fMRI activity time courses of every pair of regions, or nodes, in a brain atlas. Next, behaviorally relevant connections are identified by correlating every connection in the brain with behavior across subjects. Connections that are most strongly related to behavior in the positive and negative directions are retained for model building. A linear model relates each individual’s positive network strength (i.e., the sum of the connections in their positive network) and negative network strength (i.e., the sum of the connections in their negative network) to their behavioral score. The model is then applied to a novel individual’s connectivity data to generate a behavioral prediction. Predictive power is assessed by correlating predicted and observed behavioral scores across the group.

Because of the linear regression approach of CPM, an important aspect of the method is validation. With so many connections tested, the threshold of *p* = 0.001 (Rosenberg et al., 2016; or the threshold chosen by the researcher) during the first step may not be enough to filter through neural noise (i.e., false positives). Thus, validation is needed. According to Rosenberg et al. (2015, 2016, 2017), CPM validation could be internal or external. Internal validation requires results to be validated using a leave-one-subject-out or multi-fold cross-validation procedure. In these two procedures, significant associations between behavior and neural connectivity are identified in all subjects except ones that are left-out. A linear model is then built to best fit the relationship between network connectivity strength and the representative behavior score. Next, the left-out participant’s network strength is incorporated into the linear model and a predicted behavior score is given for the participant. This step repeats for all participants in the group. If the participants’ predicted behavior scores and original behavior scores are significantly correlated, it suggests that the connectome feature and model are able to predict novel individual’s (or the left-out subject’s) behavior scores. External validation takes a different approach using a train test method. External validation tests the connectivity of the model, built on one set of data, on a completely independent set of data. For this reason, this type of validation has been said to be more rigorous but also a more generalized approach, i.e., to be able to predict behaviors from unseen subjects without overfitting (Cohen et al., 2020; Boutet et al., 2021). These steps are illustrated in Figure 2.

Given the predictive nature of validation, CPM can also be applied to behavior prediction hypotheses. That is, a unique pattern of neural activation in associated with specific behaviors is able to predict behavior scores across different cognitive states or populations, helping to detect the cognitive state and population differences. For example, using CPM, Rosenberg et al. (2017) identified a functional brain network whose connectivity strength predicted individual differences in sustained attention performance. The identified network generalized to previously unseen individuals recruited both from the United States and China, in addition to children and adolescents. The identified network was also able to predict sustained attention scores quantified from multiple cognitive tasks designed for measuring an individual’s attention capability using both resting-state and task-evoked brain states. Together results suggest that this network may be a generalized model for sustained attention. Yet, CPM provided nuanced details by revealing novel connectivity between regions within the network which, previously, were not related to attention capability. In another example, Liu et al. (2021b) applied CPM in a more sophisticated manipulation. The experiment had participants complete challenging math problems in stressful and normal contexts. Results suggested that a resting-state network, revealed by CPM after examining the whole-brain connections, directly related to negative math performance in the stress group. The network did not successfully predict math performance in control contexts. Results suggest that stressful situations may impede the brain network’s transition from a maladaptive state in resting state to a more adaptive state during the cognitive tasks.

### Pros and Cons of Connectome Predictive Modeling

Unlike the graph theoretical approaches, CPM provides an option to investigate the relationship between brain connectivity and behaviors within specific brain regions of interest, and the whole-brain, using a data driven approach. Indeed, in conventional hypothesis-driven approaches for exploring specific cognitive functions, researchers often intuitively search for brain regions related to a cognitive function in a meta-analysis database, and then check if connectivity, or any types of network organization, in these brain regions is associated with behavioral scores. CPM provides another solution: it allows a data-driven search mechanism to discover a predictive functional network. CPM’s strength is its ability to synthesize neural and behavioral activity, making it an integral tool for cognitive and behavioral neuroscientists. However, one downfall of CPM is that the functional networks discovered may exist in the brain regions without any direct association with the task at hand. Combating this issue is still an ongoing process (Rosenberg et al., 2017). However, findings are perceived to be meaningful due to CPM’s powerful ability to predict behavior.

### Real Data Example

In our dataset, we wanted to use CPM during memory retrieval to predict memory accuracy for the wrong and correct feedback stimuli presented during the problem-solving task. Regressions between each edge in connectivity matrices during memory retrieval and behavioral memory performance scores for correct and wrong feedback stimuli were measured across n-1 subjects and used to assess the relevance of functional connections to behavior. The *p*-value in each regression between neural connectivity and behavioral outcomes (memory score for the presented feedback) was recorded in a 68 × 68 symmetric matrix (see the “Methods” section for a more detailed description of the symmetric matrix) from each frequency band, resulting in 2,244 × 4 = 8,976 *p*-values for each regression. To find the most significant associations between specific connectivity and the memory scores for both correct and wrong performance feedback stimuli, as well as to control for multiple comparisons, the resulting *p*-values were held to a 0.001 threshold (Rosenberg et al., 2016) as described above. A single summary statistic, network strength, was used to characterize each participant’s degree of connectivity by averaging all edges found below the threshold. To ensure our results pertained to positive effects on memory performance, we only involved edges in the positive tail. These edges represent a positive effect on memory performance. The identified network, constructed from the significant positive edges was then utilized for the left-out participant with both correct and wrong performance feedback stimuli to test their predictive power to memory score. This procedure repeated *n* times via a leave-one-out cross-validation to validate the network discovered.

Results show that CPM successfully identified a functional network that significantly predicted memory score for correct and wrong performance feedback stimuli, respectively, (correct: β = 0.47, *F*[1,70] = 33.13, *R*^2^ = 0.32, *p* < 0.0001; wrong: β = 0.51, *F*[1,70] = 46.30, *R*^2^ = 0.40, *p* < 0.0001). In addition, to investigate whether memory retrieval performance for correct and wrong performance feedback stimuli relies on the same functional network, networks identified in each leave-one-out round were also used to predict the memory performance for the left-out participant in the alternative memory tasks, i.e., functional networks found during the memory of correct performance feedback stimuli was used to predict the memory of wrong performance feedback stimuli, and vice versa. Results indicated that networks found in the correct performance feedback stimuli memory and networks found in the wrong performance feedback stimuli memory could not be used interchangeably for prediction, suggesting that the memory retrieval process for correct performance feedback stimuli and wrong performance feedback stimuli may rely on different connectivity, and in turn, different neural mechanisms.

## Multi-Voxel Pattern Analysis (MVPA) in Brain Networks

Multi-voxel pattern analysis (MVPA) seeks to enhance the sensitivity of detecting neural representations and cognitive states by looking at the contributions of activity from all regions of the brain (Norman et al., 2006; Mill et al., 2017). MVPA also has the capability to establish more reliable and generalized neural patterns that correspond to cognitive functions. In other words, MVPA focuses on the statistical nature of mental representations in available data and how reliably this representation can be mapped to novel and unseen data. This is exactly what machine learning techniques revolve around. Hence, MVPA is considered a technique that leverages machine learning and multivariate statistics to identify the cognitive states with distributed neural activity (Haxby et al., 2001). To achieve categorization, a common approach involves removing a part of the available data and using it to test the categorizations built on the remaining data. This train-test method is a form of cross validation. Another key step in MVPA is to establish a model that statistically describes the relations between neural representation and cognitive states in available data. This relies on advanced statistical techniques, e.g., non-linear fitting, support vector machines, artificial neural networks, and the deep learning (for details about deep learning please see *deep learning in brain networks* section).

In the first MVPA study in cognitive neuroscience (Haxby et al., 2001), illustrated that collapsing activity from multiple voxels together can be used in a well-trained model to distinguish which object categories subjects were viewing. Currently, there are many ways to conduct MVPA analyses in cognitive science. The main category of MVPA is classification (including regression which can be considered classification on continuous cognitive output) and representational learning (RSL). Although MVPA is traditionally conducted on individual neural regions, it has recently been implemented with functional connectivity and networks (Anzellotti and Coutanche, 2018).

In network-based classification, MVPA usually starts with a feature selection approach, which utilizes a *t*-test, e.g., (Wei et al., 2018) or *F*-test, e.g., (Abraham et al., 2014) that statistically tests differences between functional connectivity across the whole-brain. Significant test values are utilized to quantify areas that have the most substantial differences in connectivity. A classifier is built from these values to categorize cognitive states based on the selected connectivity. For example, Dosenbach and colleagues (Dosenbach et al., 2010) successfully separated children’s and adults’ brains using functional network MVPA in resting state. This work was further developed, leading to a new branch in neuroscience – brain age prediction. Indeed, Li et al. (2018) successfully predicted subjects’ brain age using functional network in resting-state and MVPA regression. It is worth noting that feature selection in MVPA using a data-driven approach, e.g., *t*-test or *F*-test, also faces the problem of interpretation. Results may be ambiguous. A good MVPA model not only yields good classification and prediction output, but also labels the brain representations well, i.e., the selected brain features uncovers the proper cognitive mechanisms according to the cognitive study. Indeed, Dosenbach et al. (2010) found that the weakening of short distance connections and strengthening of long-distance connections may predict brain maturity. Although vague, this result is reasonable, as the integration of more distant brain regions (through long distance connections) has been suggested to indicate more complex cognitive functions present in older humans.

Another critical MVPA approach is representational similarity analysis (RSA) (Kriegeskorte et al., 2008). RSA is a multivariate method that can be used to extract information about distributed patterns of representations across the brain (Dimsdale-Zucker and Ranganath, 2018). Rather than trying to categorize the neural representations into corresponding cognitive states like MVPA classification, RSA uses the representational distance (or, more generally, dissimilarities) between neural activity patterns associated with the cognitive states as a summary statistic to classify (Mack et al., 2013; Diedrichsen and Kriegeskorte, 2017). More generally, after all distances are measured from several cognitive states, a matrix can be constructed. From this matrix, the representational dissimilarity matrix (RDM), where the representational distance (or dissimilarity) between pairs of cognitive states can be indexed and further deciphered, can be constructed. These types of analyses are called representational geometry (Kriegeskorte and Kievit, 2013; Freeman et al., 2018).

**FIGURE 3:**
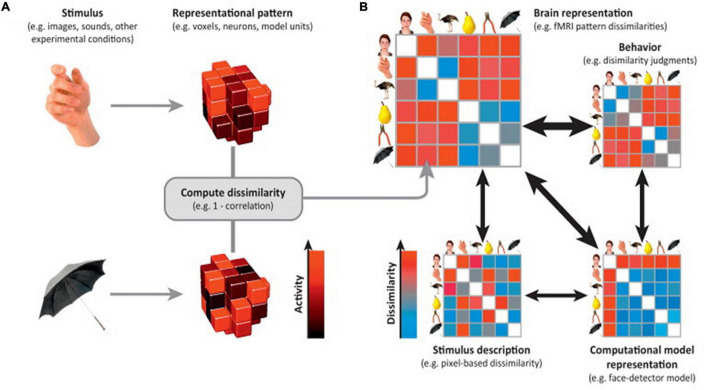
Representational similarity analysis. Illustration of the steps of RSA for a simple design with six visual stimuli. **(A)** Stimuli (or, more generally, experimental conditions) are assumed to elicit brain representations of individual pieces of content (e.g., visual objects). Here, the representation of each item is visualized as a set of voxels (an fMRI region of interest) that are active to different degrees (black-to-red color scale). We compute the dissimilarity for each pair of stimuli, for example using 1–correlation across voxels. **(B)** The representational dissimilarity matrix (RDM) assembles the dissimilarities for all pairs of stimuli (blue-to-red color scale for small-to-large dissimilarities). The matrix can be used like a table to look up the dissimilarity between any two stimuli. The RDM is typically symmetric about a diagonal of zeros (white entries along the diagonal). RDMs can similarly be computed from stimulus descriptions (bottom left), from internal representations in computational models (bottom right), and from behavior (top right). By correlating RDMs (black double arrows), we can then assess to what extent the brain representation reflects stimulus properties, can be accounted for by different computational models, and is reflected in behavior. Adapted with permission from Kriegeskorte and Kievit (2013).

In RSA analysis, the first step is to choose a brain feature (e.g., connectivity of interest) and estimate the activity pattern. The second step is to estimate the RDM. The most commonly used distance measure is the correlation distance (Pearson correlation across features selected), yet other distance measures such as the Euclidean or Mahalanobis distance can also be used. The final step is to compare the RDM to assess the extent to which different representations are alike (Aguirre, 2007; Kriegeskorte and Kievit, 2013). Depending on the cognitive states of interest, comparison of representational distance can detect different cognitive stimuli (Beaty et al., 2020), individual differences (Chen et al., 2020), neural plasticity (Fischer-Baum et al., 2017), disease abnormities (Cauda et al., 2014), longitudinal brain development (Schwartz et al., 2021), and even the representational alteration across time periods (Kobelt et al., 2021). An illustration of the technique is displayed in Figure 3.

Like MVPA classification, although RSA was first applied in decoding brain patterns in individual neural regions, it has been applied in network activity across brain regions as well. Using this approach, RSA has shown robust findings comparing fine-grained cognitive states, e.g., (Beaty et al., 2018, 2020). RSA has even been suggested to be able to identify specific individuals. In a study conducted by Finn and colleagues (Finn et al., 2015), whole-brain functional networks were utilized to extract a “brain fingerprint,” a unique neural signature, for each individual. This signature is intrinsic and can be used to identify subjects regardless of which cognitive task the subject is performing. Brain fingerprinting may represent how different individuals process a variety of cognitive tasks, including the personalized strategies, habits, or normative behaviors (Tavor et al., 2016). Using RSA, Tavor and colleagues (Tavor et al., 2016) established whole-brain functional connectivity to represent one cognitive task for every individual subject. These functional networks were then associated with a single point in representational space, such that the researcher could map the distances of all the cognitive tasks’ FC network in RDM. Results suggested that points in the RDM were closest to all other points from the same subject, no matter which cognitive state those points were related to. Each individual could be identified using the RDM.

Representational similarity analysis on network analysis can also target more refined spatial patterns by analyzing regional networks (Cole et al., 2013; Mill et al., 2017). In another memory-related study, Xue and colleagues (Xue et al., 2010) found that the subsequently remembered faces and words showed greater representational similarity in neural networks across several brain regions. This result suggested that successful memory encoding occurs when the same neural representations are more precisely reactivated across time, rather than when patterns of activation are more variable across time (Xue et al., 2010).

### Pros and Cons of Multi-Variant Pattern Analysis in Brain Networks

Instead of decoding brain patterns from activity within brain voxels or regions, using MVPA in brain networks allows cognitive states to be recognized by decoding whole-brain functional connectivity. By incorporating information from multiple connectivity networks, MVPA provides a more precise method for examining differences in small and nuanced neural activation patterns that cannot be detected using classical MVPA analysis in single brain regions. The scope of MVPA has been expanded; any techniques that manipulate the representation of distribution patterns in the brain could all be considered a variation of MVPA (Cohen, 2017). For example, RSA provides a new view for researchers to compare cognitive states in a relative way by comparing the relation between corresponding neural representations. MVPA approaches can also be used to decode dynamics in a network (Stokes et al., 2013; King and Dehaene, 2014). By evaluating the moment-to-moment variability of multivariate representations, insight into the timescale of task-related information in specific networks can be gained.

Setting up an optimal predictive model using MVPA and finding cognitively meaningful features needs to be further validated regarding neural networks. Due to the influx of advanced machine learning techniques, it is sometimes the case that researchers utilize these methods without understanding the mathematical processes behind them. Although these methods provide new resources to researchers, because of the automated nature, researchers may not be able to detect mistakes.

### Real Data Example

#### Multi-Variant Pattern Analysis-Classification

Multi-variant pattern analysis using whole-brain network connectivity was utilized to test whether memory retrieval processes for correct and wrong performance feedback stimuli could be recognized based on network connectivity. Whole-brain phase-locking values (PLV), measuring connectivity between all brain regions, were used for each participant when they were shown the veridical correct and wrong feedback stimuli during a problem-solving task. The whole-brain network in memory retrieval tasks was used as one sample in the training dataset. Classifier training and testing were conducted using a leave-one-out cross-validation approach. Classifier training began with a feature selection approach, where *t*-tests, e.g., (Cohen et al., 2016), were conducted on every single functional connectivity between correct and wrong feedback classes across the whole-brain. Connectivity with *p* < 0.05 was selected as a feature to represent the most distinct connectivity between the two groups. This procedure was conducted on all frequency bands (theta, alpha, beta, and gamma). A support vector machine (SVM) based classifier was then trained to maximally separate the two cognitive states (correct vs. wrong feedback stimuli) based on the selected connectivity. The trained classifier was then applied to classify the leave-one-out participant using the same connectivity that was selected from the training dataset.

Results exhibited that overall, only 51.2% (1,000 permutation test: *p* = 0.774) of the cognitive states were accurately classified, which means the memory retrieval for correct and wrong performance feedback stimuli can only be recognized by chance. Results from the previous section (see details in CPM section) indicate that memory retrieval processes are different between correct and wrong performance feedback stimuli; however, by using MVPA, analyses could not find direct evidence explaining how the brain performs distinctly during the two processes. This may suggest that the memory retrieval process for correct and wrong performance feedback stimuli are subject to individual differences. These results also demonstrate how choosing the correct analytic technique is integral for neural analyses. CPM provided evidence that these retrieval processes differed, whereas, MVPA demonstrated no significant differences.

To test whether the whole-brain functional network-based MVPA could be an effective process for classification, we did the same analysis to classify accurate and inaccurate memory retrieval processes, regardless of the correct or wrong performance feedback stimuli. Specifically, each participant’s memory retrieval trials where the performance feedback stimuli were accurately remembered (either correct or wrong performance feedback stimuli) were collapsed to construct a whole-brain functional network. Trials where fonts were inaccurately remembered were collapsed to construct another network. MVPA classification with leave-one-out cross-validation was applied to classify each cognitive state. Results exhibited that overall, 77.6% (1000 permutation test: *p* = 0.013) of the cognitive states were correctly classified. Together, the results suggest that accurate memory retrieval and inaccurate memory retrieval are more easily classified using brain networks than specifically classifying the correct and wrong performance feedback stimuli retrieval tasks.

#### Multi-Variant Pattern Analysis-Representational Similarity Analysis

As stated in Xue et al. (2010), memory encoding is enhanced by reactivating the initial neural representation in each subsequent study episode, and pattern reinstatement can account for subsequent memory effects in both recall and recognition tests. We hypothesize that brain pattern similarity between memory encoding and recall would be associated with the participants’ memory accuracy scores. To test this, we first constructed a PLV-based adjacency matrix for each subject in their memory encoding process and another adjacency matrix in their memory recall process. Next, a linear Pearson’s correlation was conducted between the two adjacency matrices, obtaining a *R* value to indicate the representational similarity between them. After this, another linear regression analysis was conducted between the output *R* value and the memory score overall. Results suggested marginal effects where memory scores were positively correlated with the representational similarity between encoding and retrieval for subjects for theta, alpha and beta frequency bands (Theta, β = 0.31, *F*[1,70] = 3.54, *R*^2^ = 0.055, *p* = 0.065; Alpha: β = 0.30, *F*[1,70] = 3.37, *R*^2^ = 0.051, *p* = 0.071; Beta: β = 0.28, *F*[1,70] = 3.37, *R*^2^ = 0.052, *p* = 0.070). A significant relationship was found in the gamma band (Gamma: β = 0.31, *F*[1,70] = 4.18, *R*^2^ = 0.066, *p* = 0.043). Results bolster Xue and colleagues (Xue et al., 2010) findings by suggesting that the more similar neural activity is during memory encoding and retrieval, the more accurate participants memory scores are.

## Brain Network Dynamics

Traditional analyses that test functional connectivity in the brain operate under the assumption that the activation remains constant throughout the length of the recording (Allen et al., 2012). However, the human brain operates in a more sophisticated manner. Its topological organization changes continuously, regardless of the cognitive process at hand (Tomescu et al., 2014; Chen et al., 2016; Vidaurre et al., 2018). These changes, which emerge over time scales spanning milliseconds to minutes, are non-random. Brain networks tend to exhibit a relatively stable status within a certain period of time, which is captured as a “brain state.” Thus, any brain process can be represented as a series of repeatedly emerging brain states that transition between one another in a temporally coordinated manner. Transitions between states are also non-random. Some of the brain states may play the role as a transition hub that temporally bridges other states together (Anderson et al., 2014; Taghia et al., 2018), or, a transition may only occur between specific groups of brain states (or metastates; Vidaurre et al., 2017), suggesting that brain state sequencing is temporally organized.

Brain states, and the transitions between states, occur as a result of numerous factors such as brain development, brain aging, or degenerative diseases (Wang et al., 2014). Thus, brain network dynamic analyses have been implemented in developmental work (Hutchison and Morton, 2015) and clinical diagnosis (Dadok, 2013; Ou et al., 2013; Wang et al., 2014). In cognitive tasks, there is also increasing evidence suggesting that human behavior is weighted by both spatially balanced topological patterns in brain networks and latent cognitive processes related to the networks. Different cognitive processes require the brain to change states. The brain must change from its default temporal processes, defined by a particular state, and transition to a brief, task-efficient state. Moreover, resting state conversions are also subject to individual differences. These individual differences demonstrate the value and richness in dynamic network analyses.

### Network Dynamic Construction

**FIGURE 4:**
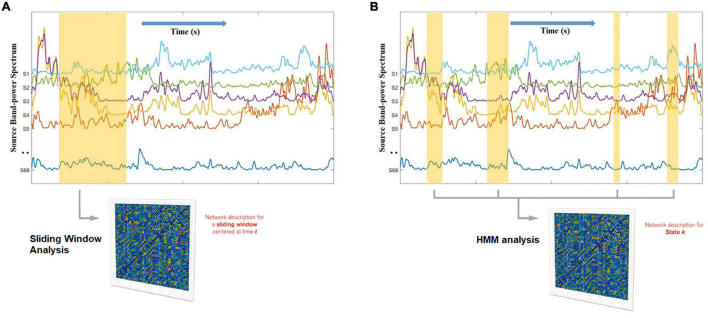
Hidden Markov Modeling (HMM) network analysis **(B)** as opposed to sliding-window network analysis **(A)**. Whereas the sliding window has a fixed width and ignores the data beyond its boundaries, the HMM automatically finds, across the entire data set, all the network occurrences that correspond to a given state, enhancing the robustness of the estimation (because it has more data than a window) and adapting to inherent network time in a data driven manner. In this example, the states themselves reflect unique spatial patterns of oscillatory envelopes and envelope couplings, that consistently repeat and different points in time. The non-marked segments of the data correspond to other states.

The most widely used approach to characterize network dynamics are sliding window (or gradually tapered) correlations between regions of interest (Di and Biswal, 2013; Kucyi and Davis, 2014; Lindquist et al., 2014; Zalesky et al., 2014). Time series data representing neural activity is input into the sliding window analysis. Connectivity within the window is computed between each pair of time series truncated by the sliding window with a Pearson correlation coefficient. Pearson correlation coefficients are calculated continuously as the sliding window moves across the time series data. When connectivity from all windows is concatenated, a set of connectivity matrices – a dynamic functional connectome, representing the temporal evolution of whole-brain functional connectivity – is obtained (Calhoun et al., 2014; Preti et al., 2017). An illustration is provided in Figure 4.

Dynamic network analyses need to be calculated in distinct ways for fMRI, EEG, and MEG methodologies. fMRI studies have a much slower time course due to the hemodynamic response in the brain. Thus, coherent oscillatory brain synchronization originating from underlying neuron activity at various frequency bands (Laufs et al., 2003; Buzsaki and Draguhn, 2004; Mantini et al., 2007) is limited. In this instance, dynamic network analyses may not accurately depict how neural states fluctuate in real time as fMRI cannot index these smaller timescales. On the other hand, methods such as EEG and MEG, which have a much higher temporal resolution in comparison to fMRI, are able to be used to estimate brain dynamics by incorporating not only cross-region temporal synchronization but also cross-region phase synchronization (Chang and Glover, 2010; Yaesoubi et al., 2015; Demirtaş et al., 2016). Cross-region and phase temporal synchronization are achieved by time-frequency analysis using short-time Fourier transformation coherence (STFT; Liu et al., 2017) or wavelet transformation coherence (Chiu et al., 2011).

### Brain States

Once the dynamic functional connectivity is established, brain dynamics can be categorized into several brain states that reoccur over time. Clustering algorithms, such as k-means clustering introduced by Allen et al. (2012, 2014) used to be the most widely used method to obtain brain states (Damaraju et al., 2014; Hutchison et al., 2014; Rashid et al., 2014; Barttfeld et al., 2015; Gonzalez-Castillo et al., 2015; Hudetz et al., 2015; Marusak et al., 2016; Shakil et al., 2016; Su et al., 2016; Liu et al., 2021a). A limitation with clustering algorithms, however, is that they summarize brain patterns only based on the spatial distribution of brain connectivity. Alternatively, Hidden Markov Models (HMMs) can be used to provide robust modeling of rapidly changing functional network structure on rapid cognitive timescales. HMM clusters brain states by simultaneously incorporating how different brain states temporally link to one another, i.e., the highest probability of how brain states were sequenced (Rabiner et al., 1989). HMM has been used in multiple studies across a range of data modalities, including fMRI (Baldassano et al., 2017), EEG (Borst and Anderson, 2015), and MEG (Vidaurre et al., 2016, 2018). An illustration is provided in Figure 4.

The functional meaning of each brain state needs to be carefully interpreted to provide a more comprehensive explanation of a cognitive process based on sequences of brain states. In brain region-based analyses, a brain state is defined using the most significantly powered region or component, i.e., a single brain region or co-activated brain regions, that explains the largest variance across all the regions (Anderson et al., 2014). For example, in an executive function study (Liu et al., 2017), one of the brain states characterized by dominant power in fronto-polar cortex was defined as the state responsible for solving problems. This was because the fronto-polar region is thought to highly correlate with cognitive processes such as reasoning and working memory (Klingberg et al., 1997; Salazar et al., 2012; Darki and Klingberg, 2015). It was also found that the longer each participant spent in this state, the more accurately they could solve the problems. On the other hand, in brain states constructed using networks and the whole-brain, there are many functional connections involved. Therefore, it is difficult to say which connections are dominant. In this case, brain states may be defined using topological organizations within the network across the brain regions (Chen et al., 2016). For example, Vidaurre et al. (2018) define brain states by finding tightly connected functional modules, while Shine et al. (2016) define brain states according to the integration/segregation of the whole-brain network.

### Brain States Features

The following five metrics are commonly assessed in brain states analyses: (1) frequency or proportional occupancy, measured as the proportion of all windows classified as instances of particular states, and computed separately for each state, (2) mean dwell time or mean lifetime, measured as the average number of consecutive windows classified as instances of the same state, (3) inter-transition interval, measured as the number of consecutive instances before a transition to the same state, (4) the number of transitions or the number of states, measured as the number of state transitions across certain conditions/individuals, which may represent the stability of whole-brain dynamics (more states means less stable and fewer states means more stable) (Liu et al., 2017), and (5) state transition probability, measured as the likelihood that a brain state at time instance t remained within the same brain state in the previous time t-1; this can be used to determine the state transition paths. For example, Taghia et al. (2018) indicated that the brain state that dominates the high-load working-memory condition does not suddenly shift to the state that dominates the fixation condition without first accessing a state associated with an intermediate cognitive demand.

### Pros and Cons of Network Dynamics

Network dynamics describe neural data unlike any other previous analysis. Neural dynamics describe how neural activation can fluctuate over time on the order of milliseconds, providing insight into how the brain networks reorganize in a temporal manner in relation to cognitive processes and behaviors. Unfortunately, because of the complexity of network dynamics, results can take a long time to compute and the results may be ambiguous. For example, Hidden Markov models need to be trained on a set of seed sequences and generally require a larger seed. This type of training involves repeated iterations of the Viterbi algorithm which can be quite slow. Moreover, the brain states that result from Hidden Markov models are defined using neural networks that are usually based on connectivity between all pairs of brain regions available for analysis. Thus, this often results in a brain state that is difficult to define, i.e., the more connections within the brain state, the more the functional definition of the brain state becomes unclear, leading to ambiguous results. Computationally, many parameters can result in overfitting, and consequently, algorithms are often unable to segment time series data effectively (Vidaurre et al., 2018). To circumvent this problem, a commonly used approach to reduce features in modeling brain states is the principal component analysis (PCA; Anderson et al., 2014; Becker et al., 2014; Vidaurre et al., 2018). Another approach proposed by Vidaurre et al. (2017) is to apply Hidden Markov models on raw region level signals instead of connectivity level signals. After characterizing the brain states, networks are estimated by pooling all data corresponding to a specific brain state. Finally, as another alternative, instead of using all connectivity, some sub-networks of interest may be pre-defined, then a single matrix representing the graph-theoretical properties of the specific sub-network can be estimated (Liu et al., 2021a). In this way, brain states can be defined based on the activity of a small number of sub-networks.

### Real Data Example

To construct dynamic functional connectivity, time series were extracted for all 68 sources in each trial, using MNE (Gramfort et al., 2013, 2014). Each memory retrieval trial was defined as the time between when the participants make their answer selections and the presentation of the performance feedback stimuli computer screen. Unlike the stationary functional connectivity calculated by phase locking, time-variant connectivity between all pairs of brain regions was generated using the spectral coherence analysis from every single EEG trial that was collected during the memory task. In other words, for each memory retrieval trial, we obtained a symmetric 68 × 68 connectivity score matrix for each time window. Each adjacent matrix was further pruned by applying a statistical threshold (Liu et al., 2011, 2013) to retain coefficients *r*_*ij*_ ≤ 30% of the total connections. We further optimized our model by examining activity over several smaller functional *subnetworks* (subsets of the entire matrix) relevant to understanding memory retrieval performance.

As discussed earlier, memory retrieval for correct fonts and wrong fronts may rely on different memory mechanisms and different brain networks. To simplify the analysis and avoid overfitting, three memory-related networks, namely the semantic memory network, the emotional memory network, and the episodic memory network, were extracted. Network strength was measured for every functional subnetwork from every time window in theta band. For every memory retrieval trial, we obtained three time-courses to represent the activity of the three functional subnetworks in terms of how closely the brain regions within each subnetwork communicate with one another compared to other nodes in the whole brain (Forbes et al., 2018) and we name it network strength.

To resolve dynamic network activity, we applied HMM to time-courses extracted from the network strength from the three functional networks. Results yielded 17 distinct brain states; the fractional occupancies (reflecting the proportion of time spent in each state) were measured for all states from every single memory retrieval trial. Then, the fractional occupancies were averaged across the correct and wrong performance feedback stimuli memory trials independently for each participant. The averaged fractional occupancies were then correlated with the memory accuracy scores (d-prime) for both correct and wrong performance feedback stimuli. Results indicated that only one of the brain states showed a significant correlation with the wrong performance feedback stimuli d-prime score (β = 2.79, *F*[1,70] = 5.23, *R*^2^ = 0.073, corrected *p* = 0.025). This state showed the highest activity or network strength in the emotional memory network, and lower activity, respectively, in the other two networks. Results suggest that the more time participants spent in this dominant state, emotional memory network, the higher the probability that they can accurately remember the wrong performance feedback stimuli from the problem-solving task. Results suggest further evidence that memory retrieval processes behind wrong performance feedback stimuli may be related to emotional processing.

## Deep Learning in Brain Network

Deep learning, or deep neural networks (DNN), is a branch of a broader family of machine learning methods. These machine learning methods are based on traditional shallower artificial neural networks (ANN) (Lecun et al., 2015; Schmidhuber, 2015). DNN significantly increase the sensitivity of conventional machine learning methods by adding more layers between input and output than ANNs (hence “deep”). Multiple layers extract different levels of representations/abstractions from the sensory input (Hinton, 2010). Recently, deep learning has become revolutionary due to its success in clinical diagnosis (Khaligh-Razavi and Kriegeskorte, 2014; Yamins et al., 2014; Eickenberg et al., 2017), image processing (Kamnitsas et al., 2017; Zhao and Mukhopadhyay, 2018; Pinto et al., 2020) and behavior prediction (Plis et al., 2014; Van Der Burgh et al., 2017; Nguyen et al., 2018).

**FIGURE 5:**
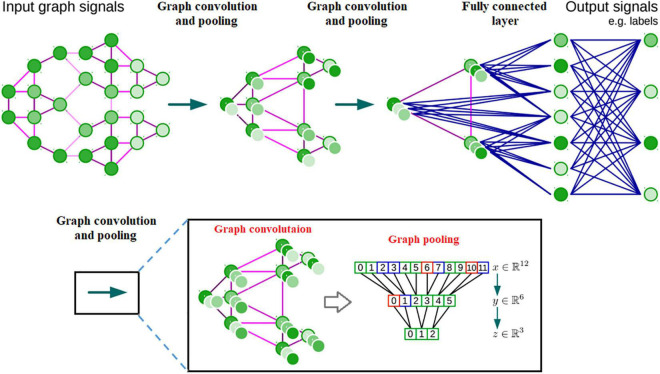
Architecture of a CNN on graphs and the four ingredients of a (graph) convolutional layer.

Brain networks, which have been widely used for exploring human brain organization and cognition (Smith et al., 2009; Assaf et al., 2010; Power et al., 2011; Yeo et al., 2011; Bertolero et al., 2015; Dubois and Adolphs, 2016; Reinen et al., 2018), unfortunately are not represented in the Euclidean grid. Instead, they are represented via graphs that depend on reciprocal relationships and similarities between pairs of brain regions. The complexity of graph data has brought significant difficulties to the application of existing DNN algorithms (Shuman et al., 2013). Recently, many studies that extend deep neural network approaches for graph data have emerged (Wu et al., 2019). The most important line of work is built on spectral graph theory (Bruna et al., 2013; Defferrard et al., 2016) which implements convolution through a complex Fourier transform on graphs. Another broad category of work relies on the graphs’ spatial information; the main idea is to generate a node’s features by aggregating its neighbors’ features, e.g., (Niepert et al., 2016; Veličković et al., 2017; Chen et al., 2018; see Figure 5 for an illustration).

Although some of the models mentioned above have been applied to brain studies (Cucurull et al., 2018; Duffy et al., 2019; McDaniel and Quinn, 2019). These works all define graphs as an architecture involving both edges and features over nodes. Importantly, they assume that the connectivity between all pairs of nodes is identical across samples (a single set of edge weights fixed for all samples). An illustration of such graph architecture can be seen in Figure 7. Additionally, a typical example of such architecture is the registered brain mesh on neural surfaces (Robbins et al., 2004; Liu et al., 2020). In contrast, the representation of whole-brain neural networks can be defined by edges between nodes, *not* features over nodes. In other words, the discrimination of brain networks for each sample lies in the connectivity strength and edge distribution between brain regions, but not the feature distribution over brain regions. Thus, the techniques adopted by most graph-based deep neural networks do not apply to actual brain network data (Kawahara et al., 2017). Therefore, to date, only a small number of studies have attempted to apply deep neural networks to brain connectivity data.

**FIGURE 6:**
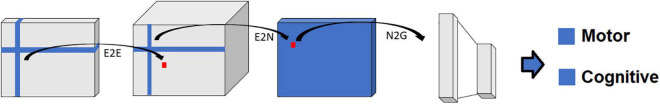
Schematic representation of the BrainNetCNN architecture. Each block represents the input and/or output of the numbered filter layers. The 3rd dimension of each block (i.e., along vector m) represents the number of feature maps, M, at that stage. The brain network adjacency matrix (leftmost block) is first convolved with one or more (two in this case) E2E filters which weight edges of adjacent brain regions. The response is convolved with an E2N filter which assigns each brain region a weighted sum of its edges. The N2G assigns a single response based on all the weighted nodes. Finally, fully connected (FC) layers reduce the number of features down to two output score predictions.

**FIGURE 7:**
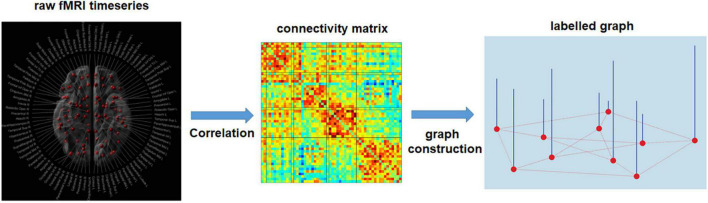
Estimation of single subject connectivity matrix and labeled graph representation. Pearson’s correlation is used to obtain a functional connectivity matrix from the raw fMRI time-series. After specifying the graph structure for all subjects, based on spatial or functional information, each row/column of the connectivity matrix serves as a signal for the corresponding node (node features). The common connectivity matrix used for all subjects can be established using the anatomical information, e.g., the spatial distance between brain regions, or the physiological information, e.g., the mean functional connectivity matrix among the training samples.

### Fully Connected Neural Network

Fully connected neural network analyses input all connectivity in a given neural network as a vector (i.e., lower triangular entries of the matrices) into a fully connected deep neural network. In this case, the model outputs are the hypothesized behavioral and demographic variables. FCNN models equally manipulate all neural connectivity. In other words, they do not take information from other neighboring connections into consideration for training. Because other neighboring connections are not taken into consideration, it creates an unbiased model.

### BrainNetCNN

Alternatively, the BrainNetCNN analyses take in connectivity matrices for input (Figure 6). Like FCNN analyses, BrainNetCNN analyses also output behavioral and demographic variables. BrainNetCNN consists of four types of layers: Edge-to-Edge (E2E), Edge-to-Node (E2N), Node- to-Graph (N2G), and a final fully connected (linear) layer. The first three types of layers are specially designed layers specific to BrainNetCNN. The final fully connected layer is the same as that used in FCNNs.

The Edge-to-Edge (E2E) layer is a convolution layer using cross-shaped filters (Figure 6) and can be considered a fundamental processing level to the sensory input. The E2N layer and N2G layer can be considered the higher level, or the abstract level, of input processing. Finally, the N2G layer outputs are linearly summed by the final fully connected layer to provide a final set of prediction values.

### Graph Convolutional Neural Network

Another technique that allows researchers to utilize whole-brain networks in deep learning is to embed neural network data into a framework such as a spectral graph convolution network (Figure 7). The critical step of this approach is to construct a standard graph structure that is representative of all subjects, in addition to assigning a feature value for each of the nodes in the brain network to represent the network differences across samples. Two approaches are applied to construct a standard graph structure (Ktena et al., 2018; McDaniel and Quinn, 2019). The first approach is based on anatomical information, i.e., the connectivity established in the common graph representing the spatial distances between connected brain regions. For the second approach, the common graph’s connectivity is estimated as the mean functional connectivity matrix among the training samples. This kind of structure is more meaningful from a neuroscientific view because it reflects the average functional connection strength between pairs of brain regions within a sample. Feature values for each of the nodes was usually assigned by nodal properties defined in graph theory, such as nodal degree, or nodal clustering coefficients.

### 3D Connectome Convolutional Neural Network

Another deep learning technique is the use of a 3D connectome convolutional neural network (CNN). Khosla and colleagues (Khosla et al., 2019) preprocessed resting state fMRI data to extract the 3D spatial structure instead of only relying on each region’s averaged information. In this study, voxel-level maps were created by analyzing each voxel’s connectivity information with respect to the averaged value of each region of interest present in the selected atlas. Using this technique allowed a specific brain region’s connectivity strength to be mapped onto the whole 3D image. The number of channels is determined by the number of regions defined in the atlas used to segment the brain. Then the problem is resolved using the classic CNN approach.

Although scientists and engineers have attempted different ways to integrate whole-brain networks into the framework of deep neural networks, the predictive power of existing models is questioned by some researchers (He et al., 2018, 2020; Khosla et al., 2019, 2021; Raviprakash et al., 2019). He et al. (2018, 2020) compared the prediction results of human cognitive performance using brain DTI structural connectivity data between the deep graph convolutional network model and a simple kernel regression model. Results indicated that the graph based deep learning models did as well or worse than the kernel regression analysis, which is a much easier model for prediction. This suggests that although deep learning has been shown to be a promising tool for neuroimaging data analysis, much more work needs to be done to verify these models in network analysis. New designs are expected in future studies.

### Pros and Cons of Deep Learning

Deep learning is the most technologically advanced method in neural data analysis. As soon as the first techniques were published, deep learning has drawn intensive attention from all scientific fields and has been very successful in some medical fields, such as medical image processing and clinical diagnosis. However, the application of deep learning approaches in cognitive neuroscience is relatively recent.

A typical deep learning model contains millions of parameters, which requires a large amount of data for training to achieve the target that researchers are interested in. This creates challenges for individual studies, where usually no more than hundreds of subjects were examined, which may not be enough to obtain a perfect model. “Big data” also creates challenges for data sharing and transparency.

Conventional deep learning approaches depend on the geometrical relevance (e.g., image voxels) of the variables within the searching field of the deep learning filters. Hence the graph-like data architectures, as represented in brain network, cannot be embedded directly into the deep learning frame. Some of the basic operations in deep learning, such as convolutions and pooling, are very difficult to realize in networks. The most widely used deep learning approaches on graphs rely on a spectral decomposition to accomplish the graph convolution. This decomposition is very ambiguous and does not provide straightforward physical meanings for interpretation. Furthermore, this approach does not focus on the topological representation (edges) of the individual network. Rather, it tends to map the feature difference in each individual node in accordance with the connections within the network. Feature representations on each node are not typically involved in a common brain network, and thus this approach cannot be precisely replicated in brain network studies. Several attempts have been made to accommodate graph deep learning to brain networks. However, these approaches appear to be either incapable to convert the connectivity and network values into truly meaningful information, or unable to provide evidence that they can describe the brain network in a proper way that relates to specific cognitive states. In summary, although deep learning has been proven to have great potential, we are still a long way away from using it to investigate whole-brain networks in an appropriate way.

### Real Data Example

In the present memory study, a GCNN approach was applied to predict memory scores in the correct and wrong performance feedback stimuli trials, respectively. To construct a fixed set of edge weights across all the participants in the two memory retrieval conditions, two 68 × 68 standard whole-brain networks were generated by averaging all functional brain networks across all subjects’ whole-brain networks (across all frequency bands). Three graph theoretical feature values (nodal degree, nodal clustering coefficient and local efficiency) across 4 frequency bands (so in total 3 × 4 = 12 feature values) were assigned over each brain region for each subject.

Graph convolutional neural networks consider spectral convolutions on graphs defined as the multiplication of a signal with a filter in the Fourier domain. The signal *h* on the graph nodes is filtered by *g* as:


$$g*h=\text{U}\left({\text{U}}^{T}g⊙{\text{U}}^{T}h\right)$$

Where *g* is a non-parametric filter defined by the N-dimensional vector of graph Fourier coefficients, where N is the number of nodes in the graph (68 in this case). Using a non-parametric filter enables the receptive field of the filter to cover the entire graph at each layer. U is the Fourier basis of the graph Laplacian L, given by the eigendecomposition of L, i.e., **L** = ***U*Λ*U^T^***, **Λ** is the ordered real non-negative eigenvalue values vector of graph Fourier transform, * is the convolution operator and ⊙ denotes element-wise multiplication. The graph Laplacian L is defined as L: = D – W where the degree matrix D is a diagonal matrix whose *i*th diagonal element *d_i_* is equal to the sum of the weights of all the edges connected to vertex i as *D*_*ii*_ = ∑_*j*_*W*_*ij*_; W is a weighted adjacency matrix encoding the connection between brain regions. After normalization, the graph Laplacian is defined as **L** = *I*_*n*_ − **D**^−1/2^**WD**^−1/2^ where *I_n_* is the identity matrix.

The output is a single value that represents the memory score for each font type. The input maps were vector-valued signals (twelve graph-theoretical features) on the graph nodes and an adjacency matrix given by the common graph architecture. The GCN used a GC8-P4-GC16-P4-FC512 architecture, where GCn is a non-parametric graph convolutional layer with n channels, P is a pooling layer and FC is a fully connected layer. Each layer is followed by ReLU non-linearity. Mean squared error (MSE) was used as the loss function with an Adam optimizer, a learning rate of 10–6, and an L2 regularization parameter of 10–8 and a batch size of 2.

Results using five-fold cross-validation indicated that prediction is poor for both correct (*r* = 0.19, *p* > 0.05) and wrong performance feedback stimuli (*r* = 0.21, *p* > 0.05) in memory score prediction, as suggested by past literature (Kawahara et al., 2017; He et al., 2020). The results suggested that even if we can construct a format to run the graph-based deep learning models on whole-brain networks, we are still far from understanding interpretations within whole-brain networks.

## Conclusion

Attributing functional connectivity and brain network activation to mental representations is challenging both theoretically and statistically. Currently, cognitive neuroscience research focuses on investigating the activation of connectivity or brain networks between brain regions that are pre-selected via hypothesis-driven approaches. In these cases, the associated ROIs are usually determined based on evidence from a narrow selection of studies in past literature, or the researcher’s own limited knowledge (see the meta-analysis section in the Supplementary Materials). Recent technical and methodological advances have initiated new strategies to navigate the neural mechanisms underlying cognitive function using multi-level, both spatially and temporally, approaches. These approaches decrease the possibility of establishing a “biased” hypothesis due to restricted or incomplete understanding of specific cognitive functions, or the possibility of generating false-positive results due to the noisy brain network representations. Multi-level methods have also provided unique neural insight into individual differences in subjects. These individual differences can be used in both clinical and social psychological applications. From a clinical perspective, individualized treatments have become popular. If individual differences can be predicted from these neural methods, primarily data-driven techniques such as deep learning, personalized treatments can be implemented on a more regular basis.

The present review describes how novel neuroscience methodologies can begin to relate whole-brain networks to cognition and behavior, both in the aggregate and over time. Graph theory and connectome-based predictive modeling provide insight into how neural architecture can change in relation to various behaviors and cognitions. Meta-analytic techniques synthesize research, bringing the scientific community closer to identifying the exact function of regions and networks. MVPA uses an entirely data-driven approach to reveal nuances behind neural activity in relation to behavior that would not be able to be seen by typical neural analyses. Furthermore, novel techniques like network dynamic modeling and deep learning allow researchers to model neural activity in a more accurate manner. Considering the brain acts as a parallel processor, researchers need to consider neural activation both over time and in layers. Network dynamic modeling and deep learning techniques allow researchers to embark on these questions.

Overall, current technology helps researchers make sense of data by employing multi-level analyses that are more accurate in modeling the whole brain as it genuinely functions. By modeling the brain in this manner, not only are researchers advancing neuroscience research by creating more accurate neural models in relation to behavior and cognition, but they are also coming closer to creating more optimal treatment options for both clinical and social psychologists.

## Author Contributions

ML and RA planned and wrote the manuscript and executed all analyses. CF provided data and edits. JS and RB provided comments and suggestions. All authors contributed to the article and approved the submitted version.

## Conflict of Interest

The authors declare that the research was conducted in the absence of any commercial or financial relationships that could be construed as a potential conflict of interest.

## Publisher’s Note

All claims expressed in this article are solely those of the authors and do not necessarily represent those of their affiliated organizations, or those of the publisher, the editors and the reviewers. Any product that may be evaluated in this article, or claim that may be made by its manufacturer, is not guaranteed or endorsed by the publisher.
