# Supplementary material for: Behavioral Studies Using Large-Scale Brain Networks – Methods and Validations
Source: Front Hum Neurosci. 2022 Jun 16;16:875201. doi: 10.3389/fnhum.2022.875201 (PMC9244405; doi:10.3389/fnhum.2022.875201)
Supplement: Supplementary file 1 [file Data_Sheet_1.docx]

**Meta-analysis**

CPM offers a completely data-driven approach for identifying the network-based neuromarkers that predict human behavior. These networks are often more robust in predicting human behaviors than other hypothesis-driven neuromarkers and graph theoretical measures (Emerson et al., 2017;Beaty et al., 2018) because of their analytic approach. However, robust neuromarkers do not necessarily indicate a clear cognitive mechanism behind behavior. Sometimes, these neuromarkers can make interpretation of these cognitive mechanisms even more ambiguous. This is because of two reasons: First, the discovered networks may be novel to the target cognitive function or behavior, i.e., previous literature may not have reported similar findings. Second, the identified regions may belong to multiple functional networks, i.e., they also interact with other regions during alternative cognitive functions. Because of the ambiguity of unfamiliar data driven networks researchers often utilize more well-defined functional networks to verify cognitive functions. In this case, a hypothesis-driven approach is still needed. Today, hypotheses can also be setup through a data-driven approach, meta-analyses, due to the dramatic rise of functional neuroimaging studies and the development of neuroimaging meta-analysis software.

There are two general approaches to neuroimaging meta-analyses: image-based meta-analyses and coordinate-based meta-analyses. NeuroVault (Gorgolewski et al., 2015) is a well-developed software for image-based meta-analyses. Because whole-brain statistical images are rarely shared (Gorgolewski et al., 2015), most meta-analytic research questions cannot be addressed with image-based meta-analysis (Muller et al., 2018). On the contrary, coordinate-based meta-analyses allow researchers to capitalize on the large number of published neuroimaging literature and provide a quantitative summary of these results to answer specific research questions. Because of the transparency of coordinate-based methods, these types of analyses are more commonly used. BrainMap (Laird et al., 2005) and Neurosynth (Yarkoni et al., 2011) are among the most utilized databases in coordinate-based meta-analyses. BrainMap relies on the relatively coarse ontology of mental functions. Neurosynth uses the automatically extracted activation coordinates for 14000+ published articles, along with the full text of those articles.

The most straightforward way to use coordinate-based meta-analyses for brain network analysis is to find a specific brain functional network and associated brain regions to represent a cognitive state. Researchers may first find the most related functional regions, and then examine if the network constructed using these regions demonstrates relationships with behavior during the cognitive task. For example, Forbes et al. (2018) utilized Neurosynth to select a number of brain regions related to emotional memory in order to conduct analyses. Findings suggested that stronger connectivity between the brain regions within this network from Neurosynth was related to stronger memory retrieval in emotional contexts. On the other hand, researchers may also directly find functional connectivity in meta-analyses to represent the cognitive state through a “co-activation map” (Laird et al., 2013), which focuses on task-dependent co-activation patterns across behavioral tasks and cognitive domains (Laird et al., 2013;Fox et al., 2014). Co-activation meta-analytic studies infer connectivity between pairs of brain regions by looking at their correlated activity in terms of how frequently the pairs of brain regions show up in public literatures with the same cognitive tasks. In other words, region-based meta-analyses survey all the brain-behavior relations reported from studies involving the same cognitive tasks, summarize them and build more comprehensive and unbiased brain-behavior relations. Co-activation meta-analyses establish new brain-behavior relationships that have never been reported in any of the individual studies they survey (Crossley et al., 2016).

Researchers may also directly utilize meta-analyses studies to explore the role of specific a brain region in terms of its interactive role or graph theoretical role in a network. This type of study can answer more broad questions about the functional organization of specific regions using “meta-connectomics” (Crossley et al., 2016). Indeed, in one meta-analysis study, Crossley et al. (Crossley et al., 2013) mapped deactivations across 110 contrasts from 67 studies in healthy subjects. Crossley and colleagues demonstrated that in the same cognitive task, the activation in one region is usually accompanied by deactivation of another region. In addition, the activation-deactivation paired brain regions usually represented the rich club-peripheral paired nodes in brain network (Crossley et al., 2013). That is, the deactivations defined the boundaries between modules, the core, and the periphery of a network in the community structure of human brain functional networks.

Another important utilization of meta-analyses is that using software such as Neurosynth allows the direct comparison between brain activation between cognitive tasks. This detail is particularly important due to the many terms researchers use across domains to describe similar concepts. For example, "anxiety" and "stress" are two terms with very close meaning psychometrically and physiologically (Vedhara et al., 2003). This overlap is also likely evident at the neural level as well. That is, the two terms yield nearly identical patterns/differences with respect to the cognitive terms. To demonstrate this with our previous example, a direct effect size-wise meta-analytic contrast was conducted between the term of "stress" and "anxiety" using Neurosynth-python. Results indicate that no significant contrasts were found in any voxels across the whole brain, indicating that stress and anxiety are highly correlated at the neural level, as well as with respect to the way in which these terms were psychometrically described in the studies included in the meta-analysis. Returning to our previous example (Liu et al., 2017), a group of subjects tried to solve math problems in a stressful environment. The study wanted to examine differences in problem solving ability in stressful and neutral (control) environments. Authors wanted to operationalize stress responses and problem-solving ability using the brain. However, because there is no designated brain area to represent stress or problem-solving, alternative terms were needed. Liu and colleagues applied interactive comparisons between several psychologically similar terms for the two classes of cognitive processes respectively, i.e. one class of stress related terms such as stress, anxiety, fear, and the other class of problem solving related terms such as working memory, attention, and executive function. As shown in Fig. 3, the most commonly found contrast was discovered in the superior-frontal cortex (to represent the problem solving process) and vACC (to represent stress or anxiety). Hence, the experiment was designed using the connectivity between superior-frontal cortex and vACC to represent the functional interaction between stressful and cognitive process such as solving difficult math problems.

Given that many terms used in cognitive studies are similar in terms of neural activity, which may cause potential risks of ambiguous interpretations, it is necessary to unify the terminologies used in cognitive studies. Tompson and Fransson (Thompson and Fransson, 2017) attempted to unify terminology for large-scale brain networks using a mass meta-analysis of fMRI data associated with networks originating from both psychology and neuroscience terms. The terms were hierarchically clustered according to their brain activation in terms of spatial similarity. The results presented are very useful as a first step in developing unified terminology for large-scale brain network studies.

INSERT FIGURE 3 HERE

To this point, we have discussed how one cognitive function can be mapped to functional connectivity among multiple brain regions based on meta-analysis. Additionally, single brain region or functional connectivity can also be associated with multiple cognitive tasks. This can be also analyzed using meta-analyses. Recently, in a novel neuroadaptive optimization design, Lorenz et al. (Lorenz et al., 2017) tried to systematically explore how distinct tasks may probe the same neural system. They used real-time fMRI in combination with modern machine-learning techniques to automatically design the optimal experiment to evoke a desired target brain state. This type of approach opens a new window for cognitive neuroscientists. If specific functional connectivity was shown to play an important role in a particular cognitive function, researchers can adaptively design personalized experiments to strengthen this connectivity. Also, due to this approach precisely targeting the functional connectivity of interest, the approach can dramatically reduce the data required for making conclusions (Lorenz et al., 2016). In another one of their studies (Lorenz et al., 2018), functional connectivity between ventral fronto-parietal network (FPN) and dorsal FPN were maximumly dissociated by adaptively selecting cognitive tasks across subjects (Fig. 4.).

INSERT FIGURE 4 HERE

**Pros and cons of meta-analyses:** One of the most significant issues facing neuroscience research is the debate over specific regions' functions. Meta-analytic techniques allow researchers to synthesize results across all neuroscience domains to make a data-driven decision about a particular region or network. Although past research has shown the importance of using meta-analytic tools, several challenges also exist regarding these analyses. In particular, the use of ambiguous terms and the inference of mental function can influence results.

It is also notoriously difficult to infer mental functions from observed brain activity, even with a large sample of data. A misleadingly narrow function may be ascribed to a brain region or network of regions which, in reality, have a broader role. For example, the function of the dACC regarding pain and other stimuli has been debated over the years. A very representative argument made from large-scale meta-analysis is from (Lieberman and Eisenberger, 2015), who claim that the “dorsal anterior cingulate cortex (dACC) is selective for pain.” This conclusion contradicts the work that suggests dACC responds to non-painful stimuli. Lieberman and Eisenberge’s conclusion resulted in another two disputes between Wager's group (Wager et al., 2016) and Liberman's group (Lieberman et al., 2016), creating numerous arguments over the exact function of this region. More rigorous methods need to be developed by detecting function in the specific brain region (De La Vega et al., 2016) so that discrepancies, such as that with the dACC, can be mitigated in the future.

**Real data example.** We hypothesized that remembering wrong performance feedback stimuli given during the problem-solving task may be more closely related to emotional memory, as suggested by (Forbes et al., 2018). Hence, two functional networks were identified via Neurosynth software; the memory retrieval network and the emotion network. Network strength, which measures brain regions' connectivity within each functional network normalized by global connectivity, was extracted from each network for each frequency band. Memory accuracy for performance feedback stimuli was regressed on network strength (for both the memory retrieval network and the emotion network) for both correct and wrong font retrieval.

Results suggested that network strength in memory retrieval networks was not predictive of the wrong or correct performance feedback stimuli memory performance. However, the emotion network was found to be positively associated with memory performance in the wrong performance feedback stimuli in the theta ($\beta= 0.74,$*F*[1,70] = 3.97, $R^{2}$=0.047, *p* = 0.030), alpha ($\beta= 0.64,$*F*[1,70] = 3.80, $R^{2}$=0.039, *p* = 0.047) and beta ($\beta= 0.55,$*F*[1,70] = 3.79, $R^{2}$=0.043, *p* = 0.021) frequency bands. In addition, network strength in the emotion network was found to be negatively associated with memory performance for correct performance feedback stimuli for all frequency bands (Theta, $\beta= -0.92,$*F*[1,70] = 5.55, $R^{2}$=0.074, *p* = 0.011; Alpha: $\beta= -0.97,$*F*[1,70] = 5.63, $R^{2}$=0.075, *p* = 0.012; Beta: $\beta= -1.20,$*F*[1,70] = 8.48, $R^{2}$=0.11, *p* = 0.0005; Gamma: $\beta= -0.93,$*F*[1,70] = 5.01, $R^{2}$=0.069, *p* = 0.027).

These results provide insight that emotional processing may be more important when remembering negative performance feedback. The presentation of the “wrong” stimuli when participants incorrectly solved a math problem may induce stronger emotion, prompting emotional memory encoding and better memory recall (higher memory accuracy scores). For accurate correct performance feedback stimuli retrieval, higher memory accuracy was related to *lower* emotional processing. This may suggest that for accurate recall of more positive performance stimuli, e.g., correct feedback related to solving a problem accurately, emotional processing is not useful. The brain regions of the two networks are listed in the Supplementary Table below (Table S1.)

Table S1.

| Memory retrieval network (left and right) | Emotion network (left and right) |
| --- | --- |
| Middle frontal cortex | vACC |
| PCC | Insula |
| Precuneus | dACC |
| Inferiorparietal cortex |  |
| Para-hippocampal cortex |  |
